# Hydrogels formed by anammox extracellular polymeric substances: structural and mechanical insights

**DOI:** 10.1038/s41598-019-47987-8

**Published:** 2019-08-12

**Authors:** Tommaso Lotti, Emiliano Carretti, Debora Berti, Costanza Montis, Stefano Del Buffa, Claudio Lubello, Cuijie Feng, Francesca Malpei

**Affiliations:** 1 Department of Civil and Environmental Engineering, Polytechnic University of Milan, Via Golgi 39, 20133 Milan, Italy; 2 Department of Chemistry “Ugo Schiff” & CSGI Consortium, University of Florence, Via della Lastruccia 3-13, 50019 Sesto Fiorentino (FI), Florence, Italy; 3 Civil and Environmental Engineering Department, University of Florence, Via di Santa Marta 3, 50139 Florence, Italy; 4 Present Address: Université de Strasbourg, CNRS, ISIS, 8 allée Gaspard Monge, 67000 Strasbourg, France

**Keywords:** Biopolymers in vivo, Environmental biotechnology

## Abstract

The recovery of biopolymers from the waste sludge produced in wastewater treatments and their application in other industrial sectors, would substantially increase the environmental and economical sustainability of the process, promoting the development of a circular economy. In this study, extracellular polymeric substances (EPS) extracted from anammox granular waste sludge, were investigated and characterized. Rheological and differential scanning calorimetry measurements on EPS aqueous dispersions indicate the formation of an extended 3-D network above a threshold concentration, with a clear dependence of the mechanical and water retention properties on EPS content. The structural characterization, performed with transmission electron microscopy and small angle X-ray scattering, reveals the presence of functional amyloids as putative structural units, observed for the first time in an EPS-based hydrogel. As a proof of concept of the applicative potential, we explored the water and grease resistance provided to paper by an EPS coating. These results shed light on the structural details of EPS-based hydrogels, and pave the way for the possible use of EPS-based materials as a cheap, eco-friendly alternative to commonly adopted paper coatings, in line with a circular economy pattern for wastewater treatment.

## Introduction

In biological wastewater treatments, microorganisms use pollutants as substrates and gain energy for growth from their transformation into harmless products. The biomass hence produced, the so-called excess sludge, is the main waste product and the related costs of handling/disposal represents up to 50% of the total wastewater treatment plant operative costs^1^. Biofilm technologies are replacing conventional activated sludge systems (CAS) because of the lower plant footprint, due to higher conversion rates and easier solid/liquid separation (e.g.^2^), and because of relevant operational costs savings, due to reduced energy consumption for oxygen supply and internal recirculation flows (e.g.^3,4^).

In biofilms, microorganisms are embedded in a matrix of hydrated extracellular polymeric substances (EPS)^5^, which overall accounts for the 50 to 90% w/w of the total organic matter^6^. Biopolymers such as EPS are gaining attention as potential alternative to conventional chemical polymers^7,8^. EPS extracted from aerobic granular sludge for instance, were recently demonstrated to bind strongly with water, to thicken or gellify liquids and to find application as flocculants or as a basis for coatings in different industrial sectors^9–11^. Moreover, EPS extraction could also improve the treatment efficiency due to the reduction of sludge volumes and the improvement of digestibility and dewaterability of the remaining sludges^12^.

Autotrophic nitrogen removal processes based on the metabolism of anaerobic ammonium oxidizing bacteria (anammox) allow removing nitrogen from wastewater with significant savings in terms of energy spent for oxygen supply (−60%), organic carbon supply (−100%) and excess sludge production (−90%)^13^. For these reasons, anammox-based technologies are rapidly emerging as the new standard for the treatment of nitrogen-rich wastewaters of municipal and industrial origins^4^, as well as key for the implementation of an overall energy-autarkic or even energy-generating sewage treatment system^14,15^. Given the fast spreading of anammox biofilm-based technology applications, and the related increasing nitrogen flux treated in these systems, anammox EPS represent a yet untapped resource to promote the desirable paradigm shift from wastewater treatment to wastewater biorefineries^2,16^.

Recent comprehensive surveys of available EPS extraction methods can be found in the literature^8,17,18^. Recently, we developed a robust chemical method for the extraction of large amount of EPS from anammox granular sludge (40% w/w) with marginal cell lysis and chemical irreversible modifications^17^. In this study we report on the production of a hydrogel composed of anammox EPS recovered from waste anammox granular sludge and water, without the addition of any cross-linker. A detailed physico-chemical characterization of the recovered biomaterial was performed, aimed at unravelling the hydrogel’s rheological and structural properties, as well as its water binding capacity, as a function of the EPS concentration, fulfilling the prerequisite for a potential application. Amyloid fibrils, observed here for the first time in anammox biofilm, were indicated as potential key elements of the complex hydrogel network. The filming properties of EPS were finally evaluated, together with the water and grease resistance effect when used as a coating agent for paper.

## Materials and Methods

### Extracellular Polymeric Substances, EPS

EPS were extracted from anammox granular sludge sampled from the full-scale anammox reactor in Rotterdam (Dokhaven-Sluisijesdijk WWTP^19^), in the period between October 2017 and February 2018. The extraction/recovery procedure was previously developed by our group and it is reported elsewhere^17^. In brief, EPS were extracted by the solubilisation of the biofilm matrix during 4 h incubation in 0.1M NaOH (10–15 mL/gTS; TS, total solids); the supernatant was recovered by centrifugation (10^4^ × g, 20 min, 4 °C) and acidified to pH 4 with 1M HCl; precipitated EPS were suspended in demineralized water with the addition of 0.1M NaOH until a final pH of around 8 was reached; the EPS dispersion was then dialyzed (3.5 kDa cut-off) against milli-Q water (3 cycles, about 3 h each) and finally freeze dried for further use of the recovered EPS. The volatile solids (VS) were measured for all fractions obtained during extraction according to standard methods^20^ (ignition for 2 hours at 550 °C), while total solids were obtained by lyophilization (VirTis, SP INdustries).

### Hydrogel preparation

The recovered biomaterial was prepared by mixing at ambient temperature the lyophilized extracted EPS and Milli-Q water in the right proportion in order to obtain the desired EPS weight fractions (5, 10, 15, 17.5, 20, 22.5, 25 wt%, gEPS gHydrogel^−1^). In order to prepare 1 gram of the biomaterial at 20 wt%, for example, 200 mg of lyophilized EPS were placed in an Eppendorf microcentrifuge tube (2 mL) together with 800 mg of Mylli-Q water; the tube was then centrifuged (1000 × g, 3 minutes, room temperature) several times (3 to 5) alternating the normal and upside down position of the tube and finally the mixture was slowly mixed manually using a glass bar until an homogeneous material was formed (ca. 30 minutes).

### Rheology

Oscillatory shear measurements were carried out with a plate-plate geometry (20 mm diameter, 400 µm gap) on a Discovery Hybrid Rheometer (Disc.HR-3, TA Instruments) working in controlled shear stress. The trend of the storage modulus (G′) and loss modulus (G″) was investigated in the linear viscoelastic region of deformations (LVR). For each EPS concentration tested, the LVR was determined through amplitude sweep (see Fig. A1 in Supplementary information for the sample containing the 20 wt% EPS, where the amplitude values of the LVR is between 0.05 and 0.5%). The G′ and G″ moduli were measured through frequency sweep tests that were carried out in the frequency range 10^−2^–10^2^ Hz at a temperature of 25.00 ± 0.01 °C with a strain of 0.4%.

The dependencies of G′ and G″ on the oscillation frequency were obtained from the phase lag between the applied shear stress and the related flow and from the ratio between the amplitudes of the imposed oscillation and the response of the gel.

The complex viscosity (|η^*^|) was calculated as:

1 $$|{\eta }^{\ast }|=\sqrt{\frac{{G}^{^{\prime} 2}(\omega )+{G}^{^{\prime\prime} 2}(\omega )}{{\omega }^{2}}}$$

where ω is the oscillation frequency in Hz.

### Differential Scanning Calorimetry, DSC

DSC measurements were performed with a Q1000 apparatus (TA Instruments). The samples, sealed in aluminum pans, were equilibrated at 25 °C, cooled to −90 °C (cooling rate = 5 °C min^−1^), kept at −90 °C for 8 min, and then heated to 30 °C (heating rate = 1 °C min^−1^) under a 50 mL min^−1^ stream of nitrogen gas. An empty sealed aluminum pan was used as the reference. For each system, three different samples were prepared and scanned. The enthalpy of fusion of water was calculated through integration of the heat flow in the temperature range from −2.8 to 6.0 °C. The Free Water Index (FWI), a parameter commonly used to represent the amount of free and bound-freezable water contained in the samples, was calculated using the following formula^21^:

2 $${\rm{F}}{\rm{W}}{\rm{I}}={{\rm{\Delta }}H}_{{\rm{s}}{\rm{a}}{\rm{m}}{\rm{p}}{\rm{l}}{\rm{e}}}{/{\rm{\Delta }}H}_{{\rm{f}}{\rm{r}}{\rm{e}}{\rm{e}}{\rm{w}}{\rm{a}}{\rm{t}}{\rm{e}}{\rm{r}}}\cdot 100$$

where ΔH_sample_ is the enthalpy change due to the fusion of the ice contained in the EPS hydrogel sample (J g_water_^−1^), experimentally determined from the DSC curve; ΔH_freewater_ (333.6 J g^−1^) is the theoretical value of the specific fusion enthalpy of pure ice at 0 °C.

### Samples preparation for transmission electron microscopy, TEM

EPS hydrogel at 25% wt% was fixed with glutaraldehyde (2.5%), p-formaldehyde (4%) and cacodylate buffer (0.1M). After this step, samples were washed with the same cacodylate buffer three times, for 10 min each wash. Then, the sample was fixed with a solution containing cold 2% osmium tetroxide (OsO_4_), 5 mM calcium chloride and 0.8% potassium ferrocyanide in 0.1M cacodylate buffer. All suspensions were washed twice with cacodylate buffer 0.1M for 10 min, and once with distilled water, for 10 min. Samples were stained with 5% uranyl acetate for 1 h, washed three times with distilled water for 10 min and dehydrated in a series of increasing strength of acetone solutions (50%, 2 × 70%, 95%, 2 × 100%) for 15 min each step. Fixed EPS hydrogel was then infiltrated with three increasing concentrations of Epon LX 112 resin in acetone (2:1; 1:1; 1:2), ending in 100% resin overnight. All samples were embedded in Epon LX112 embedding medium at 60 °C for three days. Embedded samples were trimmed and sectioned on an Ultracut E-Reichert-Jung ultramicrotome. Thin sections (100 nm thickness, approximately) taken from each sample and retrieved to copper grids were allowed to dry, and then stained with 5% uranyl acetate for 35 min plus lead citrate for 1 min. These grids were examined with a Hitachi H-300 electron microscopy using Kodak 4489 electron microscopy film.

### Atomic Force Microscopy, AFM

AFM was performed in non-contact mode, by means of a XE-7 microscope (Park Instruments) equipped with NCHR probes, using a cantilever’s resonant frequency of 300 kHz and a set-point of 15–20 nm, depending on the sample. To acquire AFM images, few drops of a 0.02 and 0.001%wt EPS solution in water were deposited on a mechanically exfoliated mica substrate, then the specimen was rinsed with acetone, dried in air and fixed with a double-sided adhesive tape under the microscope.

### Small-Angle X-ray Scattering, SAXS

SAXS spectra were acquired at the SAXS beamline of synchrotron radiation Elettra (Trieste, Italy) operated at 2 GeV and 300 mA ring current. The experiments were carried with 𝜆 = 1.5 Å and SAXS signal was detected with Pilatus 3 1M detector in the 0.008 Å^−1^ to 0.45 Å^−1^ Q-range (Q being the scattering vector), the sample to detector distance was 130 cm^22^. Samples were prepared as described in paragraph 2.2, SAXS curves were recorded at r.t. in a sample-holder for pastes. Each sample was measured 20 times for 3 seconds each time, to check for radiation damage. Data reduction and analysis of SAXS curves were carried out with Igor Pro (Wavemetrics, Portland, OR, USA).

### Water and grease resistance of EPS-based coatings

Paper sheets preparation, coating, spreading and water/oil-grease permeability tests were performed at Innovhub-SSCCP (The Italian Pulp and Paper Research Institute, Milan, Italy). For the production of paper sheets, long and short cellulosic fibers (30/70%, g/g) were previously treated using the Valley Beater method (ISO 5264-1:1979) with refining duration of 40 minutes, resulting in 38 Schopper Riegler degrees (°SR). Paper sheets were prepared according to standard methods (UNI EN ISO 5269-2:2005) with a drying time of 420 seconds at a temperature of 92 °C, resulting in squared sheets (15 × 15 cm) with a weight of 37.3 g/m^2^. Two different aqueous coating solutions were prepared: with carboxymethyl cellulose (CMC) (Finnfix CMC by CP Kelko, Atlanta GA, USA) only (10% g/g-solution) and with a mixture of CMC and EPS (10% CMC and 4% EPS g/g-solution, respectively). CMC coating solution had a viscosity of 108.8 mPa·s (Brookfield Viscometer, 25 °C, 100 RPM). The paper sheets coated with CMC only were used as experimental control. The coating spreading was performed with the automatic film applicator Sheen 1137 (Sheen Instruments, Cambridge, UK) using a Mayer coating rod (1120/25/76 mm) at a rod speed of 100 mm/s. The amount of coating solution applied on 15 × 15 cm paper samples was 0.21 and 0.26 grams using the prepared CMC only and CMC + EPS coating solutions, respectively. The coating solution application resulted in a surface specific dried coating dosage of 9.3 and 11.7 g/m^2^ for CMC only and CMC + EPS (8.31 g/m^2^ of CMC and 3.39 g/m^2^ of EPS), respectively. The resulting weight of coated paper samples after drying were 38.2 and 38.9 g/m^2^ for CMC and CMC + EPS, respectively. Water absorbance capacity was measured according to the Cobb method (UNI EN ISO 535:2014 method). The paper surface wettability was characterized by the evolution in time of the contact-angle and the volume of a water drop (analysis performed according to the standard measuring procedure ATICELCA MC 21-72). The resistance to grease, oil and waxes was evaluated by measuring permeability (turpentine oil method, ISO 16532-1:2010) and surface repellency (ISO 16532-2:2010 method).

## Results and Discussion

From a chemical point of view, EPS is composed of several species, namely polysaccharides, proteins (structural proteins or exoenzymes), nucleic acids, (phospho-) lipids, humic substances and intracellular polymers from lysed cells^8,23^. In water, these different components can interact to form complex supramolecular assemblies. When mixing EPS and water in different proportion, a visual inspection revealed a marked viscosity enhancement, as the weight fraction of EPS increased (0.5–30 wt% range tested).

**Figure 1:**
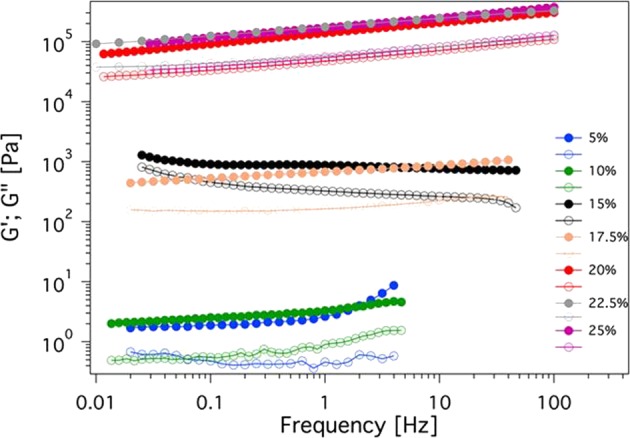
Trend of the elastic modulus *G*′ (full symbols) and of the viscous modulus G” (open symbols) as a function of the oscillation frequency of the applied shear stress for the investigated EPS based systems (amplitude strain, γ: 0.4%).

To gain more insight on the rheological properties, a series of EPS/H_2_O samples were investigated through frequency sweep curves. Figure 1 shows the trend of the storage modulus G′ and the loss modulus G″ at a constant strain of 0.4% as a function of frequency for all of the investigated systems. The storage modulus is always larger than the loss modulus, and no crossover between the G′ and G″ profile is observable within the accessible range of frequencies. This behavior (G′ > G″ all over the investigated frequency range) is typical of systems that have an infinite relaxation time τ. Being gels classified as fluids characterized by a τ value that tends to infinite^24^, the trend observed accounts for the gel nature of the EPS-based systems. Furthermore, *G*′ does not practically depend on frequency, and changes from about 20 Pa up to 20000 Pa by increasing the EPS concentration, while the rheological behavior remains almost unchanged.

**Figure 2:**
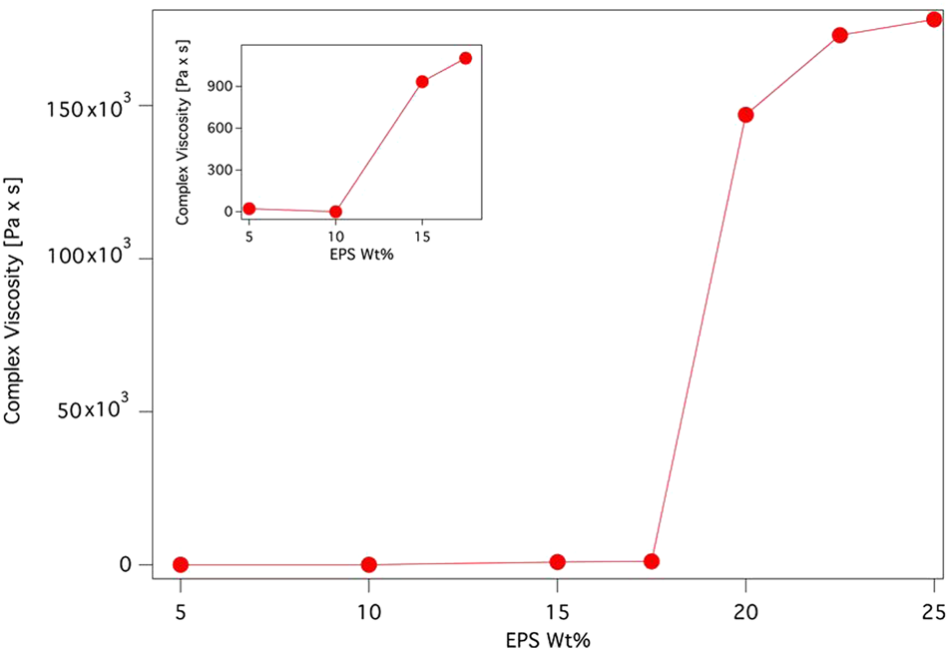
Trend of the complex viscosity, η*, as a function of the EPS concentration. The insert is a magnification of the Complex Viscosit values for the samples with EPS concentration 5, 10, 15 and 17.5 wt% EPS.

The complex viscosity η* (Fig. 2), extracted from the frequency sweep curves at oscillation frequency ω = 1 Hz, is nearly constant (in the order of few tens/hundreds of Pa·s) up to ca. 17.5 wt% EPS, and then abruptly increases to reach 23 · 10^3^ Pa·s at C* = 20 wt% EPS, indicating the occurrence of a concentration threshold which marks the formation of a an extended 3D hydrogel network.

**Figure 3:**
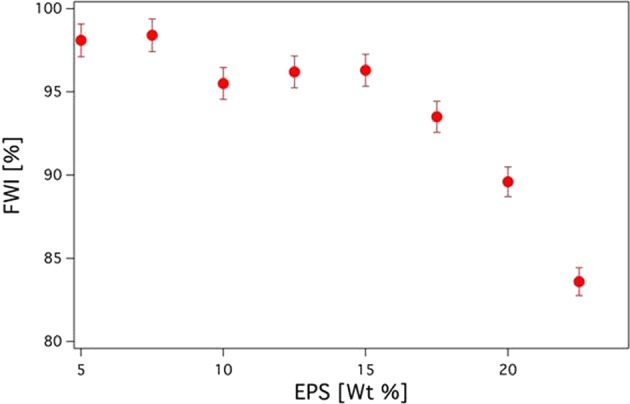
Trend of the Free Water Index determined through DSC measurements for EPS-based aqueous systems as a function of the EPS content.

Differential Scanning Calorimetry (DSC) was performed to infer the Free Water Index (FWI), determined for all the analyzed samples and shown in Fig. 3. Above 17 wt% of EPS a rather steep decrease of the FWI can be appreciated (from 97% for 15 wt% EPS, down to 85% for 20 wt% EPS), in line with the trend of the complex viscosity. This finding indicates that when a 3D network is formed, H_2_O molecules are tightly bound to the EPS matrix^25^, most likely through hydrogen bonding interactions, and are not able to freeze anymore. Interestingly, this water binding capacity would negatively affect dewatering, commonly applied in the waste sludge handling facilities. These results support previous reports on the negative effect of increasing EPS concentration on sludge dewaterability^26^.

Based on the rheological behavior, further hypothesis can be formulated regarding the structure of the EPS hydrogel. As the storage modulus *G*′ results to be proportional to the entanglement density *ρ*_*E*_ (*G*′ = *ρ*_*E*_*K*_*B*_*T*, where *K*_*B*_ is the Boltzmann constant and *T* is the temperature (K))^27,28^, an increase of *ρ*_*E*_ with the EPS content indicates a higher complexity of the sample’s structure and an increase of the strength of the formed 3D network.

The dependence of *G*′ on the weight fraction of the gelling agent *ϕ* has been largely discussed in the literature^29^. In most cases, the trend is described by a power law function, *G*′ = aϕ^n^^30^, as in this case. Figure A2 (Supplementary information) shows the variation of the *G*′ values at *ω* = 1 Hz for EPS based systems as a function of the wt% EPS. The power-law fits satisfactorily the experimental data, resulting in a *n* value of 11.1. For solid-like systems that, as in this case are constituted by permanent crosslinks between the polymeric chains (called also “frozen hinged junctions”), the Jones and Marquès theory^31^ describes the relation between the elastic modulus *G*′ and the network volume fraction C (assumed here as approximable to the weight fraction, wt% EPS) as follows: *G*′ ∝ C^(3+D^_F_^)/(3−D^_F_^)^ where *D*_*F*_ is the fractal dimension of the objects that gives an indication of the complexity of the structure of the investigated object at the observation scale. For instance *D*_*F*_ is equal to 1 for straight fibrils such as *e.g*. agarose gel fibrils. Then, in this case, the calculated fractal dimension *D*_*F*_ is equal to 2.50 indicating that the network is probably constituted by interconnected branched fibrillar units^30^.

**Figure 4:**
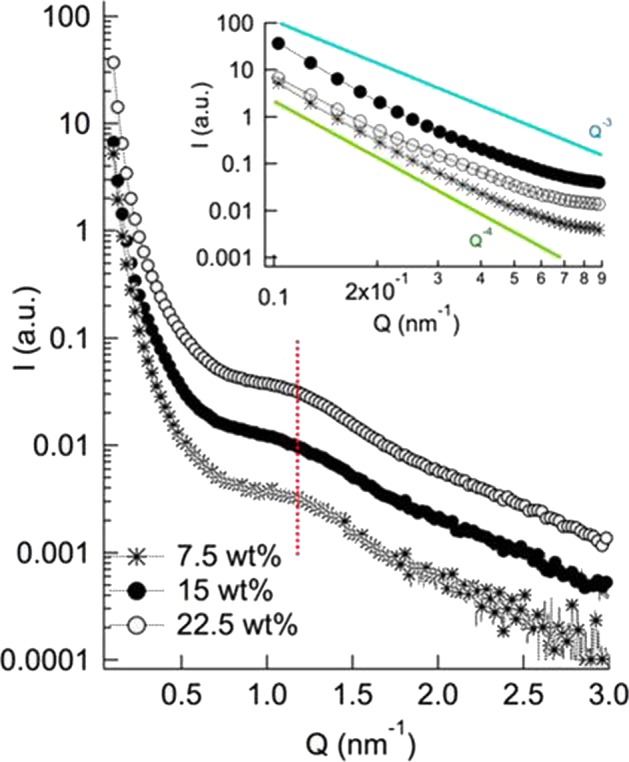
SAXS profiles of EPS/water samples with increasing amounts of EPS (7.5, 15, 22.5 wt%); (inset) magnification of the low Q region, with the dependence of the scattered intensity from Q^−4^ highlighted.

In order to gather information on the structural units constituting this 3D network, we performed Small Angle X-ray Scattering (SAXS). Figure 4 shows the SAXS profiles of the hydrogels containing different concentrations of EPS (namely, 7.5, 15 and 22.5 wt%).

Despite the complexity of the material, extracted from a biological matrix, SAXS profiles are characterized by very well-defined features. First, in the low Q region a power law trend, *i.e*., $I(Q)\propto {Q}^{-4}$, can be observed in all the three samples. This Q^−4^ dependence of the scattered intensity is a typical signature of the Porod region of solid materials, corresponding to the scattering of 3D mass fractals, observed for instance in xerogels^32^.

Therefore, irrespectively of the concentration of EPS, the network is characterized by structural units with a typical dimension higher than the experimental resolution (roughly 50 nm), which, at this lengthscale, appear compact. We can hypothesize that these structural units at a higher (micron) lengthscale, form the 3D network with 2.5 fractal dimension as highlighted from rheology.

In addition, a clear hump can be detected for the three samples, centered around Q = 1.2 nm^−1^, (corresponding to a distance of 5.3 nm in the direct space). The specific and invariant Q-position of the hump suggests that it is due to a precise structural motif, which persists as the concentration is increased without modifications in Q position but only in intensity (see also 2D SAXS images in Fig. A3 in the Supplementary information). Therefore, we can hypothesize that this feature is not due to a characteristic of the 3D network of the system (that is, it is not due to a correlation distance between different objects constituting the network, which would be expected to decrease as the concentration is increased), but it is rather due to defined structural motifs of EPS matrix, whose scattering intensity increases as their weight fraction is increased. To further address this point, the samples were imaged with Transmission Electron Microscopy (TEM).

**Figure 5:**
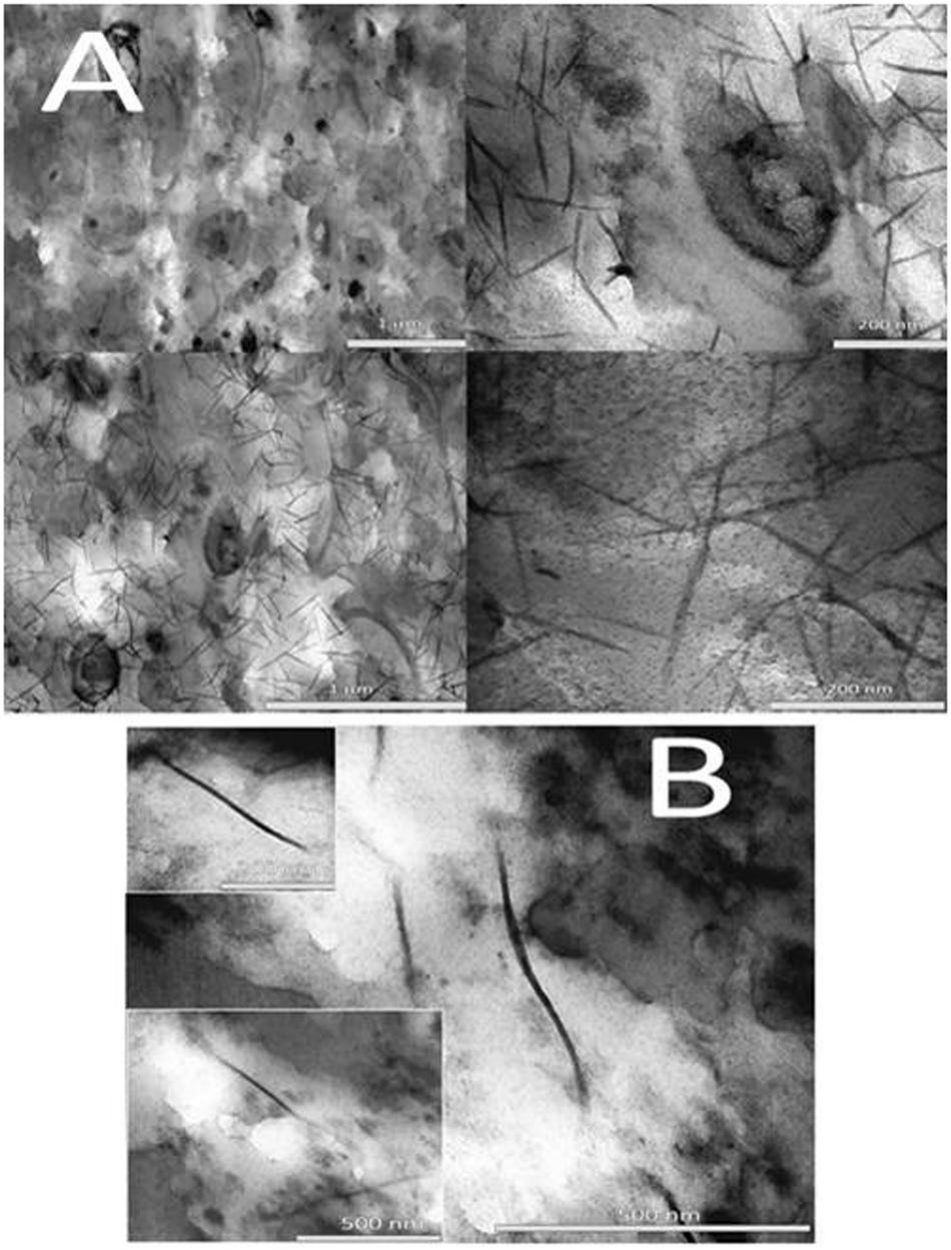
TEM electron micrographs of (**A**) natural granules and of (**B**) EPS hydrogel 25 wt% (the thickness of the thin sections is 100 nm).

Figure 5A reports some representative TEM images of thin slices of the native granule. Interestingly, a main component of the EPS is constituted by fibrils, which are also present in the EPS hydrogel upon extraction and treatment (Fig. 5B). A size analysis of the fibrils in the native granule reveals that they are characterized by a relatively highly monodisperse width (around 10 ± 1 nm, n.25) and more polydisperse length (200 ± 50 nm, n.25) (see Fig. A4 in Supplementary information for details). The fibrils in the extracted EPS (particularly in the 25% w/w gel), show no major structural variations. Possibly a slight elongation can be inferred, probably due to extraction and re-constitution of the hydrogel which allows prolonged contact between the fibrils: this effect is not observed in the native granule due to the presence of the solid matrix with slower translational kinetics and contact between separated fibrils. The observed dimensions and shape are compatible with functional amyloid fibers found in several natural biofilm matrices^33,34^. Furthermore, the anammox EPS used in this study were recently characterized in terms of protein secondary structure by means of spectroscopic methods (Circular Dichroism, Fourier Transform InfraRed and the benzothiazole dye Thioflavin-T staining technique coupled with fluorescence spectroscopy) which results indicated the presence of amyloid-like aggregates^17^.

Functional amyloid fibers, observed here for the first time in anammox granules and extracted EPS (Fig. 5), have compatible dimensions as those detected in SAXS for all the EPS gels^35^; fibril-fibril interaction might be, at high EPS percentages, implied as structuring agent^36^ in the gel-like properties of the material as highlighted from rheology.

Despite the complexity of the material, this study provides a first rheological and structural characterization of the system, which is a necessary prerequisite for potential applications of the recovered bio-based material. Insight in the structural components of anammox biofilm matrix is also essential for the development of applied wastewater treatment processes. In fact, the slow growth rate of anammox bacteria (i.e. bottleneck kinetic) impose the maintenance of very long solids retention time (SRT) in engineered systems, which is mostly accomplished by the implementation of biofilm-based technology such as granular sludge^4^. Knowledge on the EPS components responsible for the cohesiveness and mechanical properties of anammox biofilms is therefore of paramount importance in order to control undesired biofilm detachment/breakage resulting in detrimental biomass washout.

Particularly interesting is the presence in high amounts of amyloid fibrils, both in the EPS and in the derived gels. In recent years there has been a growing interest in the development of functional materials based on non-pathogenic amyloids. In particular, naturally occurring amyloids such as functional amyloids^34^ or amyloids obtained from the induced-amyloidization of natural proteins (as β-lactoglobulin) under controlled environmental conditions have been exploited for the delivery of nanosized iron^37^, for water purification^38^ and for sensing^39^. In our case, the viscous and high water-retentive material, can be of possible interest for applications in the agricultural/chemical fields and as rheology conditioner/emulsion stabilizer in the field of formulations.

**Figure 6:**
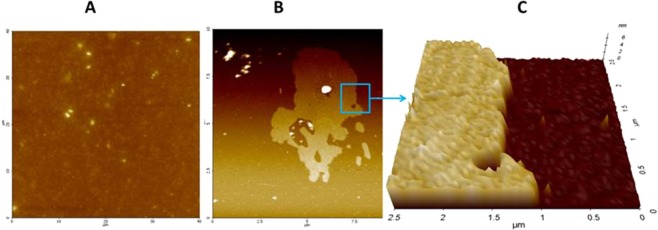
Representative AFM images of solvent-casted EPS-based films on mica substrates. (**A**) 0.02% w/v aqueous solution, 40 × 40 µm^2^ scan, Z-scale = 0–100 nm; (**B**) 0.001% w/v aqueous solution, 10 × 10 µm^2^ scan, Z-scale = 0–20 nm; (**C**) 3D image zoom of the portion delimited by the blue square.

Moreover, preliminary tests indicated that it is possible to obtain uniform films by drying EPS extract solutions. AFM study has been carried out on some films obtained by drying EPS extract solution (few drops of a 0.02 and 0.001%wt EPS solution in water) on the surface of mica specimens, in order to obtain information about their morphological properties (Fig. 6).

The film obtained from a 0.02% w/v solution (Fig. 6A) resulted in the formation of a highly uniform film, characterized by a low roughness (root-mean-squared roughness of about 7 nm) and by the ability to completely cover the mica substrate in a homogeneous manner. With a more diluted solution, only µm-sized patches of EPS are observed, with no homogenous coverage of the underlying substrate (Fig. 6B). These features resulted to be about 6 nm thick (Fig. 6C), with a roughness of about 4 nm. Such filming properties could be of interest from applicative standpoints and could be exploited for the development of coating/sizing agents in the paper and/or textile industry^40^.

In order to exploit the film ability of EPS, its applicative potential as coating agent for paper industry was tested. Particular attention was paid to the EPS ability to form barrier and to act as repellant for grease, oil and waxes. A solution of EPS 4 wt% in the presence of carboxymethyl cellulose (CMC) 10 wt% is spread onto the surface of a sheet of paper. CMC that is used as viscosity modulator for the application of the EPS based coating on the surface of paper was applied together with EPS in order to confer to the solution a viscosity (108.8 mPa·s measured with a Brookfield Viscometer at 100 RPM at 20 °C) suitable for a reproducible and homogeneous coating/spreading by automatic film applicators. The expected thickness of the CMC + EPS coating is in the order of some tens of microns. The effect of the CMC + EPS coating, was compared with a coating solution prepared with only CMC (10 wt%), used as experimental control. The analysis was also performed on pristine paper samples for comparison. Results demonstrate that the application of EPS as coating agent for paper translates into interesting water barrier properties, as well as resistance to grease penetration and absorbance. In particular, CMC + EPS coating reduced the water absorbance on the coated side by 30% compared to the blank and control samples, with an important reduction of the 34 and 23%, respectively, also on the back side (Table A1 in Supplementary information). These results are confirmed by the wettability tests (Fig. A5,A in Supplementary information). It’s interesting to note that the contact angle for the CMC + EPS coated paper is lower than the uncoated paper (blank), while is slightly higher than the CMC-coated paper (control) (Fig. A5,B in Supplementary information). This suggest that EPS limits the increase in water spreading favored by CMC coating, while noticeably decreasing water absorbance and therefore acting as a water barrier. The grease resistance of EPS-based coating was evaluated by a simple standard test. The time taken for a simulated “fat material” (*i.e*., palm kernel oil) to penetrate (break-through) the paper sheet increased from 30 s (CMC coating) to 120 s for CMC + EPS, showing the capability of EPS-based coating to reduce grease permeability. The grease resistance effect exerted by EPS was partly confirmed by the increase of the degree of surface repellency observed for the CMC + EPS coated paper with respect to CMC coating and, to a larger extent, to the untreated paper (Table A2 in Supplementary information). The capability of EPS to be adsorbed on paper cellulosic fibers and act as barrier to water and grease penetration is very interesting and can be attributed to the filling/masking of the pores of the paper and to the ability to decrease the surface energy. Given the complex and heterogeneous composition of EPS, the exact mechanism causing this enhancement of the repellant power to hydrophobic substances and water is laborious to define and should be the focus of future dedicated studies.

## Conclusions

In this contribution we have shown that extracellular polymeric substances extracted from anammox granular waste sludge are able to form hydrogels with solid-like mechanical properties, without any additional chemical cross-linker. Rheological and DSC analysis showed that for EPS concentrations above 17% w/v an extended 3D network is formed conferring to the material high mechanical and water retention properties. The structural characterization of the material suggest that functional amyloids represent the structural units of the 3D network formed in concentrated EPS dispersions. Interestingly, diluted EPS dispersions can be successfully used to produce homogenous films with extremely low roughness. As a proof of concept, the application of EPS as a coating agent on paper greatly improves the water and grease resistance of the treated substrate. The reported properties of EPS-based materials could open new scenarios for the exploration of its potential applications as a resource in the context of circular economy.

## Supplementary information

Supplementary Information

## References

[1] Kroiss H (2004). What is the potential for utilizing the resources in sludge?. Water Sci. Technol..

[2] Van Loosdrecht MCM, Brdjanovic D (2014). Anticipating the next century of wastewater treatment. Science.

[3] Bengtsson S, de Blois M, Wilén B-M, Gustavsson D (2018). Treatment of municipal wastewater with aerobic granular sludge. Crit. Rev. Environ. Sci. Technol..

[4] Lackner S (2014). Full-scale partial nitritation/anammox experiences - an application survey. Water Res..

[5] Flemming H-C, Wingender J (2010). The biofilm matrix. Nat. Rev. Microbiol..

[6] Flemming H-C, Neu TR, Wozniak DJ (2007). The EPS Matrix: The “House of Biofilm Cells”. J. Bacteriol..

[7] More TT, Yadav JSS, Yan S, Tyagi RD, Surampalli RY (2014). Extracellular polymeric substances of bacteria and their potential environmental applications. J. Environ. Management.

[8] Seviour T (2018). Extracellular polymeric substances of biofilms: suffering from an identity crisis. Water Res..

[9] Lin Y, De Kreuk M, van Loosdrecht MCM, Adin A (2010). Characterization of alginate-like exopolysaccharides isolated from aerobic granular sludge in pilot-plant. Water Res..

[10] More TT, Yan S, Tyagi RD, Surampalli RY (2016). Biopolymer production kinetics of mixed culture using wastewater sludge as a raw material and the effect of different cations on biopolymer applications in water and wastewater treatment. Water Environ. Res..

[11] Sam SB, Dulekgurgen E (2016). Characterization of exopolysaccharides from floccular and aerobic granular activated sludge as alginate-like-exoPS. Desalination Water Treat..

[12] van der Roest HF, van Loosdrecht MCM, Langkamp EJ, Uijterlinde C (2015). Recovery and reuse of alginate from granular Nereda sludge. Water.

[13] Hu Z (2013). Nitrogen Removal by a Nitritation-Anammox Bioreactor at Low Temperature. Appl. Environ. Microbiol..

[14] Kartal B, Kuenen JG, van Loosdrecht MCM (2010). Sewage treatment with anammox. Sci..

[15] Lotti T, Kleerebezem R, Lubello C, van Loosdrecht MCM (2014). Physiological and kinetic characterization of a suspended cell anammox culture. Water Res..

[16] Puyol D (2017). Resource Recovery from Wastewater by Biological Technologies: Opportunities, Challenges, and Prospects. Front. Microbiol..

[17] Lotti T (2019). Extraction and recovery of structural extracellular polymeric substances from anammox granular sludge. J. Environ. Management.

[18] Feng C, Lotti T, Lin Y, Malpei F (2019). Extracellular polymeric substances extraction and recovery from anammox granules: Evaluation of methods and protocol development. Chem. Eng. J..

[19] van der Star WRL (2007). Startup of reactors for anoxic ammonium oxidation: experiences from the first full-scale anammox reactor in Rotterdam. Water Res..

[20] Eaton, A. D. American Public Health Association (APHA), Standard Methods for the Examination of Water and Wastewater. 21st edition, APHA-AWWA-WEF, Washington, D.C. (2005).

[21] Shibukawa M, Aoyagi K, Sakamoto R, Oguma K (1999). Liquid chromatography and differential scanning calorimetry studies on thestates of water in hydrophilic polymer gel packings in relation to retention selectivity. J. Chromatogr. A.

[22] Baglioni M, Montis C, Chelazzi D, Giorgi R, Berti D (2018). Polymer Film Dewetting by Water/Surfactant/Good‐Solvent Mixtures: A Mechanistic Insight and Its Implications for the Conservation of Cultural Heritage. Angew Chem Int Ed Engl..

[23] Flemming HC (2016). Biofilms: an emergent form of bacterial life. Nat. Rev.-Microbiol..

[24] Almdal K, Dyre J, Hvidt S, Kramer O (1993). Towards a phenomenological definition of the term ‘gel’. Polymer Gels and Networks.

[25] Carretti E, Matarrese C, Fratini E, Baglioni P, Dei L (2014). Physicochemical characterization of partially hydrolyzed poly(vinyl acetate)–borate aqueous dispersions. Soft Matter.

[26] Li XY, Yang SF (2007). Influence of loosely bound extracellular polymeric substances (EPS) on the flocculation, sedimentation and dewaterability of activated sludge. Water Res..

[27] Gottlieb M, Macosko CW, Benjamin GS, Meyers KO, Merrill EW (1981). Equilibrium Modulus of Model Poly (Dimethylsiloxane) Networks. Macromolecules.

[28] MacKintosh F, Käs J, Janmey P (1995). Elasticity of Semiflexible Biopolymer Networks. Phys. Rev. Lett..

[29] De Gennes PG (1976). Scaling Theory of Polymer Adsorption. J. de Physique.

[30] Guenet JM (2000). Structure versus Rheological Properties in Fibrillar Thermoreversible Gels from Polymers and Biopolymers. J. of Rheol..

[31] Jones JL, Marques CM (1990). Rigid polymer network models. J. de Physique.

[32] Pizzorusso G (2012). Physicochemical Characterization of Acrylamide/Bisacrylamide Hydrogels and Their Application for the Conservation of Easel Paintings. Langmuir.

[33] Larsen P, Nielsen JL, Otzen D, Nielsen PH (2008). Amyloid-like adhesins produced by floc-forming and filamentous bacteria in activated sludge. Appl. Environ. Microbiol..

[34] Erskine E, MacPhee CE, Stanley-Wall NR (2018). Functional Amyloid and Other Protein Fibers in the Biofilm Matrix. J. Mol. Biol..

[35] Oliveira CLP (2009). A SAXS Study of Glucagon Fibrillation. J. of Molec. Biol..

[36] Jung JP, Gasiorowski JZ, Collier JH (2010). Fibrillar peptide gels in biotechnology and biomedicine. Pept. Sci..

[37] Shen Y (2017). Amyloid fibril systems reduce, stabilize and deliver bioavailable nanosized iron. Nat Nanotechnol..

[38] Bolisetty S, Mezzenga R (2016). Amyloid-carbon hybrid membranes for universal water purification. Nat. Nanotech..

[39] Li C, Adamcik J, Mezzenga R (2012). Biodegradable nanocomposites of amyloid fibrils and graphene with shape-memory and enzyme-sensing properties. Nat Nanotechnol..

[40] Lin YM, Nierop KGJ, Girbal-Neuhauser E, Adriaanse M, van Loosdrecht MCM (2015). Sustainable polysaccharide-based biomaterial recovered from waste aerobic granular sludge as a surface coating material. Sustain. Mater. and Technol..

