# Supplementary material for: Hydrogels formed by anammox extracellular polymeric substances: structural and mechanical insights
Source: Sci Rep. 2019 Aug 12;9:11633. doi: 10.1038/s41598-019-47987-8 (PMC6690907; doi:10.1038/s41598-019-47987-8)
Supplement: Supplementary file 1 — Supplementary Information [file 41598_2019_47987_MOESM1_ESM.pdf]

Hydrogels formed by anammox extracellular polymeric substances: structural and mechanical insights.

Tommaso Lotti[a,c,*], Emiliano Carretti[b], Debora Berti[b], Costanza Montis[b], Stefano Del Buffa[b†], Claudio Lubello[c], Cuijie Feng[a], Francesca Malpei[a]

[a] Department of Civil and Environmental Engineering, Polytechnic University of Milan, Via Golgi 39, 20133 Milan, Italy

(E-mail: tommaso.lotti@unifi.it, cuijie.feng@polimi.it, francesca.malpei@polimi.it)

[b] Department of Chemistry "Ugo Schiff" & CSGI Consortium, University of Florence, Via della Lastruccia 3-13, 50019 Sesto Fiorentino (FI), Florence, Italy

(E-mail: emiliano.carretti@unifi.it, debora.berti@unifi.it, montis@csgi.unifi.it)

[c] Civil and Environmental Engineering Department, University of Florence, Via di Santa Marta 3, 50139 Florence, Italy

(E-mail: tommaso.lotti@unifi.it, claudio.lubello@unifi.it)

†Present affiliation: Université de Strasbourg, CNRS, ISIS, 8 allée Gaspard Monge, 67000 Strasbourg, France (E-mail : delbuffa@unistra.fr)

[*]**Corresponding author**

Supplementary information

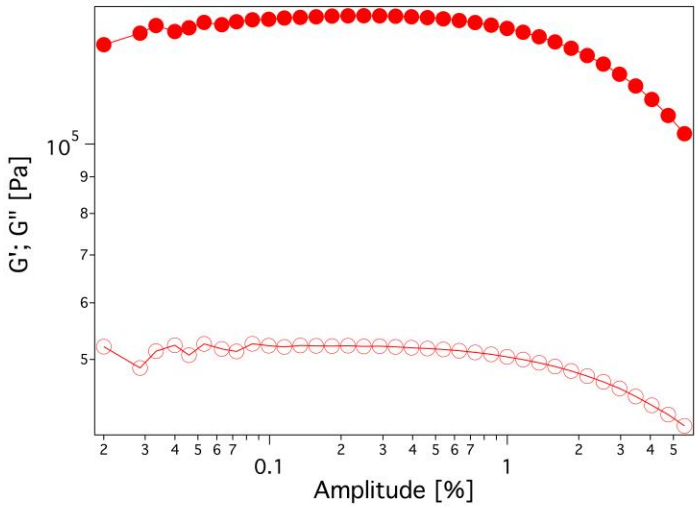

Figure A1. Amplitude sweep curve of the 20 wt% EPS based system (oscillation frequency, ω: 1 Hz).

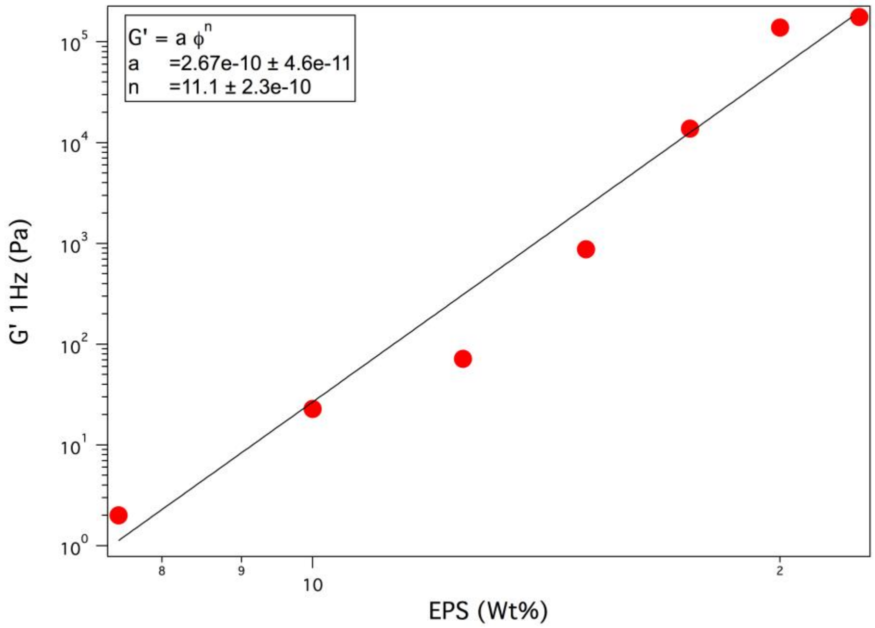

Figure A2. Double-logarithmic scale representation describing the effect of the concentration of EPS network on the value of the elastic modulus G’ at ω=1 Hz. The line represents the best linear fitting.

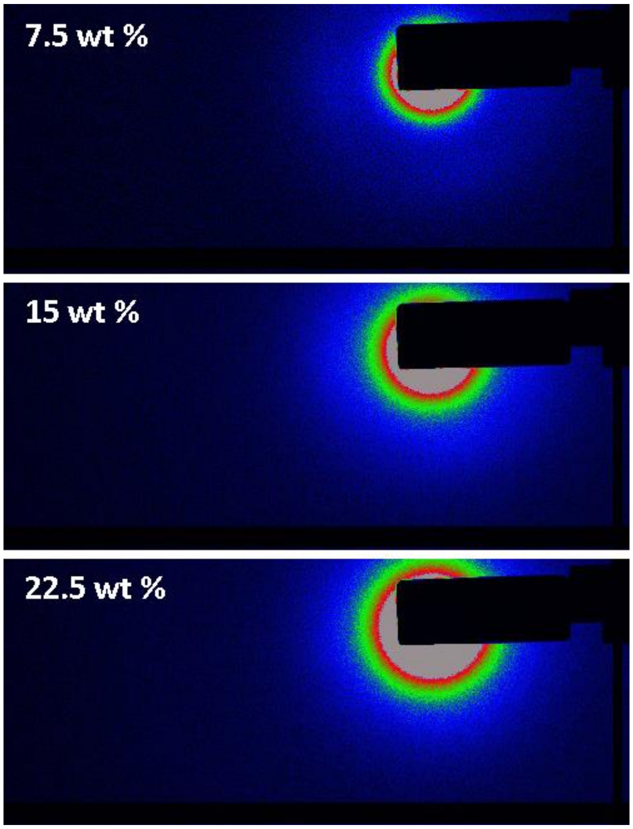

Figure A3. SAXS raw data: detector 2D images of of EPS/water samples with increasing amounts of EPS (7.5, 15, 22.5 wt%).

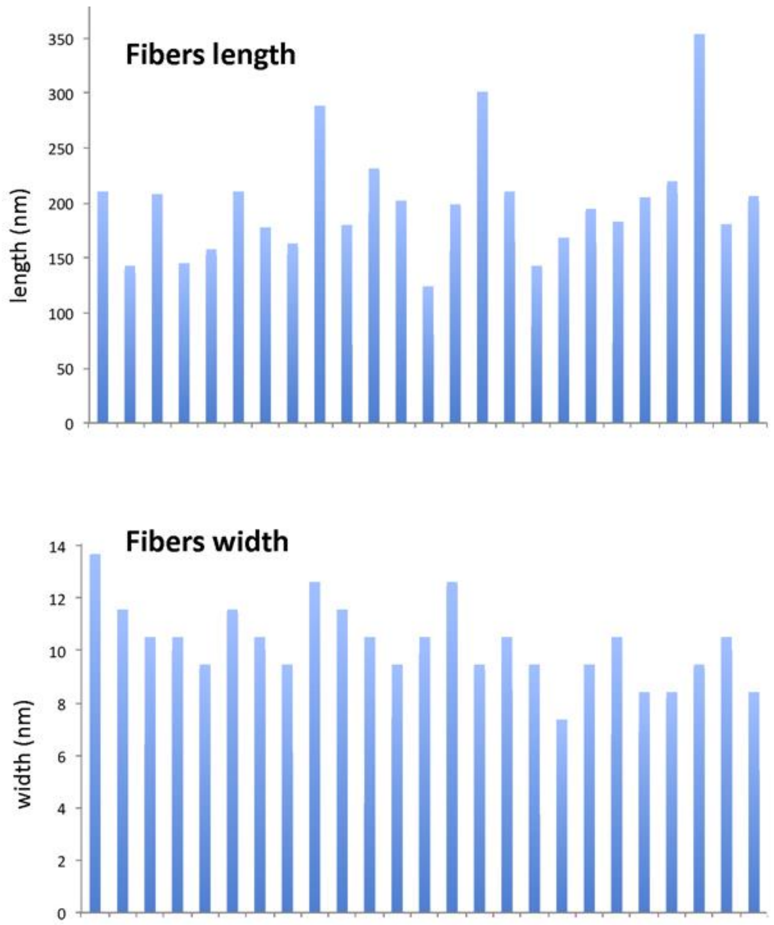

Figure A4. Size analysis of fibers in the native globule: length (upper panel) and width (lower panel) of 25 different fibers, from 5 different TEM images where evaluated. Image J was employed for size analysis. The resulting estimated values are: fibers length 200 ± 50 nm, fibers width 10 ± 1 nm.

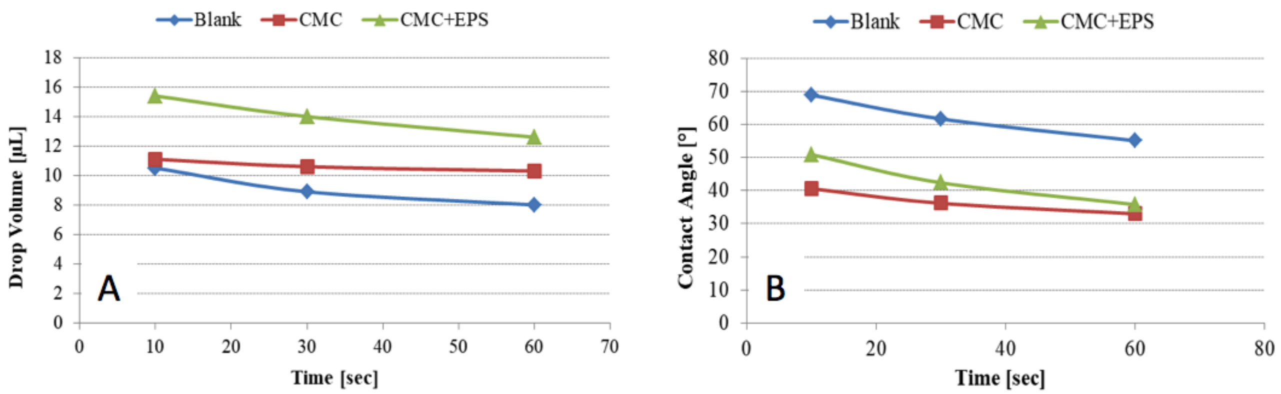

Figure A5. Evolution in time of the volume (left) and contact angle (right) of a water drop deposited on paper sheets untreated (Blank) and coated with CMC or CMC/EPS mixture.

Table A1. Water absorptiveness (Cobb method).

Sample	Coating	Water absorbance [g/m^2]	
		Coated side	Back side
Blank	none	45.3	60.3
Control	CMC	43.0	51.8
Test	EPS+CMC	31.5	40.0

Table A2. Grease resistance in terms of permeability and surface repellency.

Sample	Coating	Permeability [sec]	Surface Repellency Degree	
			Coated side	Back side
Blank	none	< 30	1	1
Control	CMC	30	3	3
Test	EPS+CMC	120	5	3
